# Hedgehog Signaling: Linking Embryonic Lung Development and Asthmatic Airway Remodeling

**DOI:** 10.3390/cells11111774

**Published:** 2022-05-28

**Authors:** Ling-Hui Zeng, Muhammad Qasim Barkat, Shahzada Khurram Syed, Shahid Shah, Ghulam Abbas, Chengyun Xu, Amina Mahdy, Nadia Hussain, Liaqat Hussain, Abdul Majeed, Kashif-ur-Rehman Khan, Ximei Wu, Musaddique Hussain

**Affiliations:** 1 Department of Pharmacology, Zhejiang University City College, 51 Huzhou Street, Hangzhou 310015, China; zenglh@zucc.edu.cn; 2 Key Laboratory of CFDA for Respiratory Drug Research, Department of Pharmacology, School of Medicine, Zhejiang University, Hangzhou 310058, China; qasimbarkat@zju.edu.cn (M.Q.B.); xuecheng@zju.edu.cn (C.X.); 3 Department of Basic Medical Sciences, School of Health Sciences, University of Management and Technology Lahore, Lahore 54000, Pakistan; shahzadakhrram543@gmail.com; 4 Faculty of Pharmaceutical Sciences, Government College University, Faisalabad 38000, Pakistan; shahid.shah@gcuf.edu.pk (S.S.); ghulamabbas@gcuf.edu.pk (G.A.); liaqathussaingsk@yahoo.com (L.H.); 5 Medical Pharmacology Department, International School of Medicine, Istanbul Medipol University, Istanbul 34000, Turkey; amasallam@medipol.edu.tr; 6 Department of Pharmaceutical Sciences, College of Pharmacy, Al Ain University, Al Ain 64141, United Arab Emirates; nadia.hussain@aau.ac.ae; 7 Faculty of Pharmacy, Bahauddin Zakariya University, Mulatn 60000, Pakistan; abdulmajeed@bzu.edu.pk; 8 Faculty of Pharmacy, The Islamia University of Bahawalpur, Bahawalpur 63100, Pakistan; kashifurrehman@iub.edu.pk

**Keywords:** hedgehog signaling, lung development, asthmatic airway remodeling, targets pathogen, repairment in tissue and immune system

## Abstract

The development of the embryonic lung demands complex endodermal–mesodermal interactions, which are regulated by a variety of signaling proteins. Hedgehog (Hh) signaling is vital for lung development. It plays a key regulatory role during several morphogenic mechanisms, such as cell growth, differentiation, migration, and persistence of cells. On the other hand, abnormal expression or loss of regulation of Hh signaling leads to airway asthmatic remodeling, which is characterized by cellular matrix modification in the respiratory system, goblet cell hyperplasia, deposition of collagen, epithelial cell apoptosis, proliferation, and activation of fibroblasts. Hh also targets some of the pathogens and seems to have a significant function in tissue repairment and immune-related disorders. Similarly, aberrant Hh signaling expression is critically associated with the etiology of a variety of other airway lung diseases, mainly, bronchial or tissue fibrosis, lung cancer, and pulmonary arterial hypertension, suggesting that controlled regulation of Hh signaling is crucial to retain healthy lung functioning. Moreover, shreds of evidence imply that the Hh signaling pathway links to lung organogenesis and asthmatic airway remodeling. Here, we compiled all up-to-date investigations linked with the role of Hh signaling in the development of lungs as well as the attribution of Hh signaling in impairment of lung expansion, airway remodeling, and immune response. In addition, we included all current investigational and therapeutic approaches to treat airway asthmatic remodeling and immune system pathway diseases.

## 1. Introduction

The adult human lung is a tree-like structure consisting of branches of airway coils connected to a single tube, the trachea. The latter delivers breathed air through bronchi and bronchioles into the highly vascularized gas exchange units, the alveoli. The lung is among the human organs that possess a low steady-state cell turnover yet can enormously respond to repair damaged cell safter injury [1]. For the provision of moisture to the air and protection from pathogens, the airway epithelium forms a thin liquid surface layer comprising glycoproteins and mucins. The airways are lined by two types of epithelial cells such as ciliated and secretory cells along with club cells (SCGB1A1+) [1]. Club cells are self-renewing progenitor cells that can develop into ciliated cells. Subsequently, the airway eventually terminates and quickly moves into the distal alveoli, which are small air vesicles made up of alveolar epithelial cells (AEC) type 1 and type 2. AEC1s occupy over 90% of the alveolar surface in adults and regulate respiration between the alveoli and the blood [2,3]. Surfactant proteins are produced by AEC2s to sustain alveolar fluid balance, and surfactant protein C (SFTPC) is recognized as a specific marker for AEC2s. In order to maintain alveolar homeostasis, AEC2s can self-renew and differentiate into AEC1s. Different mesenchymal cell types in their particular stem cell components regulate the characteristics of club cells and AEC2s, and they have the unique ability to give feedback to progenitor cells in their immediate vicinity [4].

**Figure 1:**
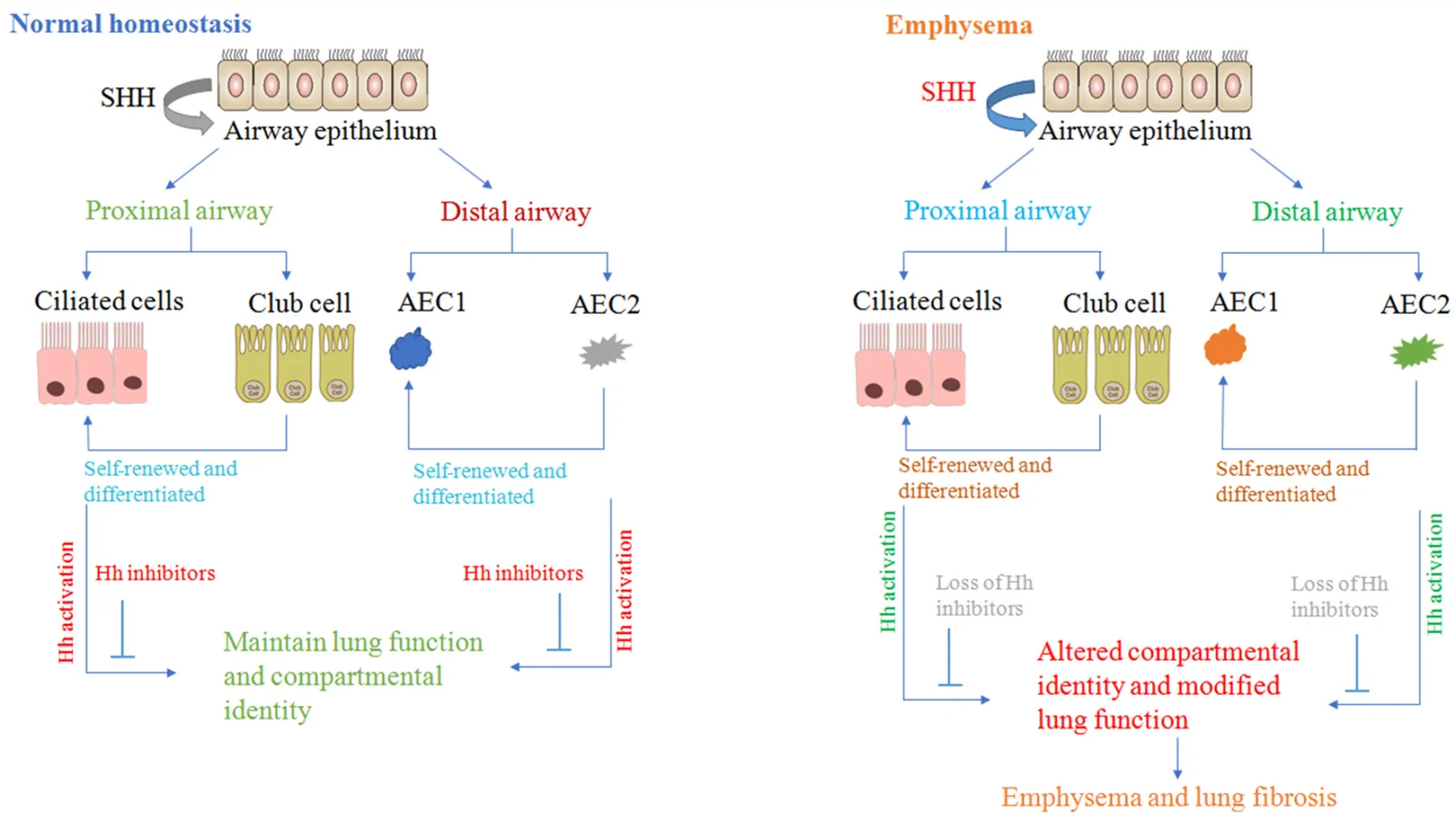
Asymmetric hedgehog activation model that maintains distinct compartmental identity. We propose that differential Hh activation is a promising mechanism for maintaining compartment-specific identity as well as function in the lungs. Endogenous inhibitors of Hh activation may be lost, disrupting the physiological asymmetry of Hh signaling and resulting in altered compartmental identity and the structural changes observed in lung disorders. Adapted from Wang et al., *J. Dev. Biol.* 2019, 7, 14 [4].

Hedgehog (Hh), the family of a morphogenic protein, is one of the highly regulated signaling pathways, which is significant in organogenesis, proliferation, and differentiation [5,6]. In insects, Hh controls the proper segmentation and maturation of wings [7], whereas in vertebrates, it induces left-right asymmetric embryonic development as well as regulates the accurate development of the lungs, nervous system, muscles, skin, skeleton, eyes, and intestine, and the differentiation of cartilage and sperms [6,8]. Sonic (Shh), Indian (Ihh), and Desert (Dhh) Hedgehog are three Hh ligands found in animals [9]. In adult humans, Hh maintains the stem cells and progenitor cells within various organs, such as the skin, brain, bladder, prostate, and other organs [10,11], and also controls tissue regeneration and repairing. Furthermore, it also regulates aging and body height, as well as participates in chronic degenerative and inflammatory diseases [12]. The proximal airway and distal alveolar epithelium both express Shh, which is the most common ligand in the adult human lung [13,14]. The Hh-receptive compartment is made up of GLI2+ lung mesenchymal cells, which may be found in both the proximal and distal mesenchyme. Surprisingly, the proximal airway mesenchyme has stronger Hh signaling activity than the distal alveolar mesenchyme, as demonstrated by the presence of Hh target genes (GLI1, Ptch1, and Ptch2) in the proximal mesenchyme [13]. The asymmetric Hh activation is illustrated in Figure 1.

Deactivation of Hh signaling pathway may lead to genetic developmental defects, i.e., holoprosencephaly, whereas mutation-induced over-expression of this pathway may result in cancer of various organs, including the lungs, breast, pancreas, and prostate [15,16]. Moreover, the activated Hh pathway is also associated with pulmonary, hepatic, and renal tissue fibrogenesis [17]. Lung development depends on particular organization of the Hh signaling pathway, while an altered pathway induces numerous neonatal respiratory disorders such as asthma, chronic obstructive pulmonary disease (COPD), and lung cancer, all of which are associated with airway remodeling [18].

**Figure 2:**
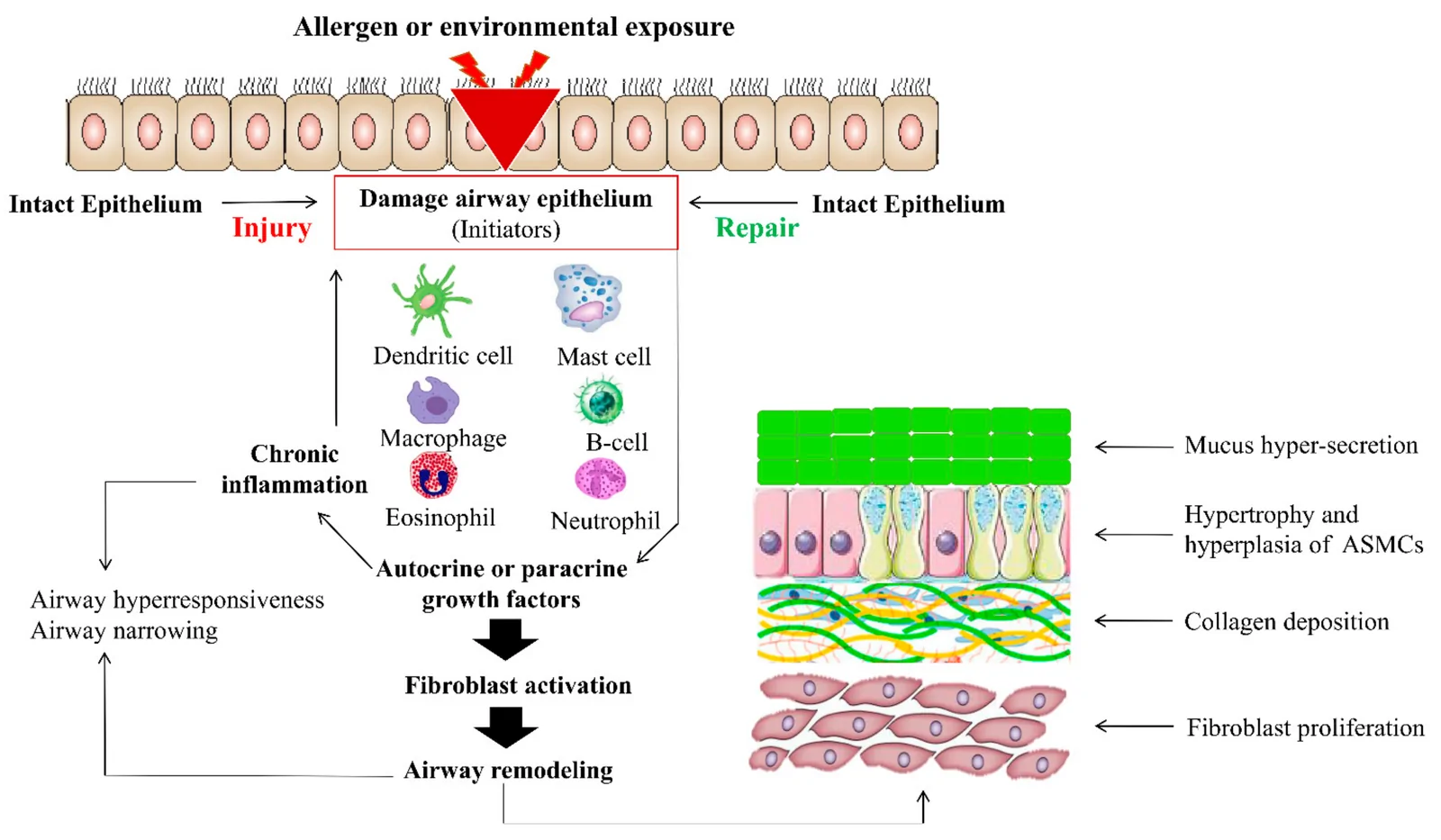
Airway remodeling. Allergens or environmental exposure damage the airway epithelium, which leads to activation of immune cells through releasing soluble factors. Airway epithelial and inflammatory cells contribute to the production of autocrine and paracrine factors that induce the proliferation, expansion, and activation of airway smooth cells, which ultimately initiate the morphogenesis in the lung development. Consequently, they produce a suitable environment for sustaining the chronic inflammation that causes the airway remodeling.

Asthmatic airway remodeling, described for the first time in 1922, is characterized by irreversible structural changes in the airway wall [19]. Chronic inflammation and disruption of the airway repairing system during asthmatic disorders lead to the emergence of airway remodeling, which is simply an irreversible alteration in the airway wall. Such asthmatic remodeling is characterized by thickening and the fibrosis of subepithelial basement membrane and the accumulation of fibroblasts, myofibroblasts, fibrogenic growth factors, and the extracellular matrix in the sub-epithelial region of proximal airways [20,21,22,23]. Airway remodeling also results in goblet cells metaplasia (mucus production), hypertrophy, and hyperplasia of airway smooth muscle cells (ASMCs), as well as angiogenesis [23,24,25]. The epithelial–mesenchymal transition (EMT), categorized by the loss of the epithelial phenotype by bronchial epithelial cells and the acquisition of a mesenchymal phenotype, also contributes to airway remodeling by acting as a source of fibroblasts as well as by secreting extracellular matrix (ECM) molecules [26]. Airway remodeling mediates the pulmonary dysfunction and infiltration of inflammatory cells accompanied by cytokines, chemokines, and growth factors under the impact of different key proteins, commonly known as morphogens, such as Notch, Wnt/β-catenin, sonic hedgehog, and epidermal growth factor [18]. The airway remodeling in the lungs is presented in Figure 2.

We propose that differential Hh activation is a promising mechanism for maintaining compartmental-specific identity as well as function of the lungs. When endogenous inhibitors of Hh activation are lost, they can disrupt the physiological asymmetry of Hh and result in the altered compartmental identity and structural changes observed in lung disorders.

In this study, we discuss the molecular basis and current investigations linked with Hh signaling and its role in lung organogenesis. We also present the contribution of abnormal stimulation or removal of Hh signaling to the induction or development of pulmonary alteration, along with its influence in linking embryonic lung development and asthmatic remodeling and immune diseases. Finally, all current investigational and potential therapeutic approaches used to treat airway asthmatic remodeling and repair immune system pathway diseases are highlighted.

### 1.1. Regulation of Hedgehog Signaling

The vertebrate Hh signaling pathway is composed of the following major constituents: (a) three different Hh ligands, including Shh, Ihh, and Dhh; (b) trans-membrane patched receptors (Ptch), having two isoforms encoded as Ptch-1 and Ptch-2; (c) smoothened receptors (Smo-A, Smo-B, and Smo-C); and (d) a cytoplasmic complex that controls the transcription factors, including Cubitus interruptus (Ci) in insects or the GLI family of the zinc-finger DNA-binding domain in vertebrates (GLI-1, GLI-2, and GLI-3) [27,28]. Additionally, negative regulators (Hh inhibitor protein that competes with the Hh ligand for binding to Ptch) and positive regulators (growth arrest-specific 1, CAM-related/downregulated by oncogenes (CDO), and brother of CDO) are also present on the cell surface and decrease and increase the binding of ligands to Ptch, respectively [9,29]. Shh is a major ligand that regulates the morphogenesis of many organs [30], while Ihh and Dhh have a role in the development of bone and nerve sheaths, respectively [31,32].

**Figure 3:**
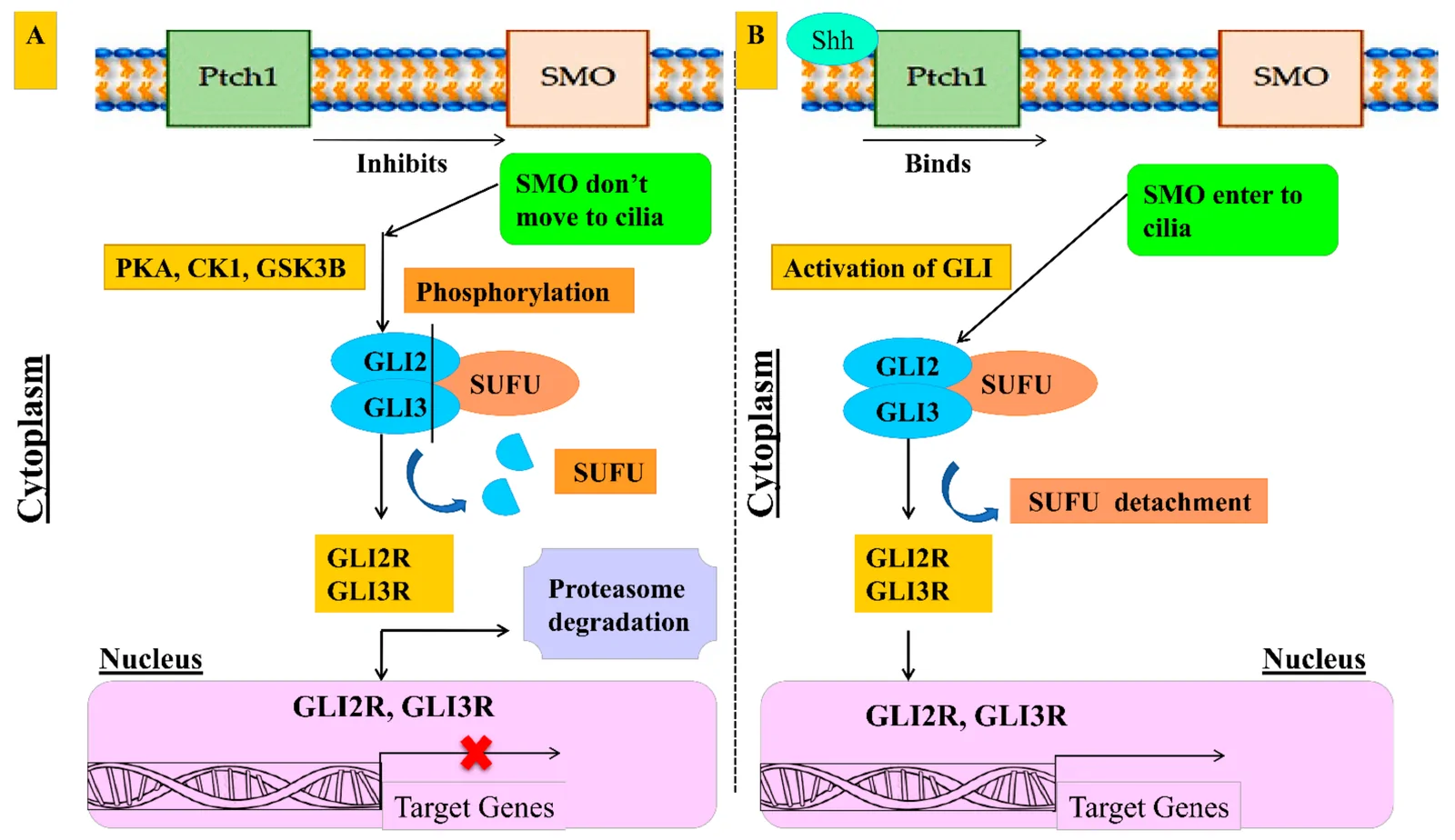
Sonic hedgehog signaling pathway(**A**) In the absence of the Shh ligand, Patched-1 (Ptch1) inhibits the Smoothened receptor (SMO), and SMO cannot enter the primary cilium. The phosphorylation of GLIS can be phosphorylated by the complex of glycogen synthase kinase 3b (GSK3b), protein kinase A (PKA), and casein kinase 1 (CK1). The phosphorylated GLI-2R and GLI-3R were formed into two possible destinations: proteasome degradation and nucleus translocation to suppress the transcription of target genes. (**B**) Hedgehog proteins bind to Ptch1 on the cell surface level, and SMO enters to primary cilium to activate GLI through Suppressor of fused (SUFU) detachment. Then, GLI-2R (mostly) andGLI-3R translocate to the nucleus.

Hh signaling is propagated by the binding of the Hh ligand to Ptch and Smo receptors. In the absence of the Hh ligand, the transmembrane Ptch blocks the formation of active Smo-C from transmembrane Smo-B (inactive Smo-A and Smo-B remain in equilibrium via retrograde intraflagellar transport; IFT), while in the presence of the Hh ligand, Smo gets activated, which results in cascade of events dealing with the translocation and binding of GLI with DNA, via the zinc-finger domain, where GLI transmits transcriptional information in response to the Hh ligand. The GLI family acts as a transcriptional activator or repressor. GLI-2 and GLI-3 contain activator (carboxyl-) and repressor (amino-) terminals, respectively [29,33], whereas GLI-1 acts as an activator, as it contains only the carboxyl-terminal; thereby, GLI-1 is the transcriptional target and reporter of Hh signaling [34]. GLI-3 performs primarily as a repressor, whereas GLI-2 works as a major pathway activator. Ultimately, the equilibrium between GLI-2A and GLI-3R results in nuclear transcription, which activates the required target output. The Hh signaling pathway in the absence or presence of the Shh ligand is depicted in Figure 3.

In the absence of the Hh ligand, the pathway is divided into two separate processes: (1) GLI-2/GLI-3 undergo the phosphorylation by CKI, PK-A, and GSK-3β, and ubiquitination for partial proteasomal cleavage (through E-3 ligase) into truncated repressor forms, primarily for GLI-3, which is converted to GLI-3R, and to a lesser extent for GLI-2, by elimination of activation terminal (carboxylic) domain, thereby exhibiting the partial degradation of both or complete degradation of GLI-2 [35]; (2) the GLI transcriptional activator is inhibited by the suppressor of fused homolog (SUFU) [36]. Primarily, GLI-3R translocates to the nucleus, seemingly by IFT, and inhibits the transcription of the target gene. Upon Hh signaling activation, Ptch moves out of the cilium and is degraded by the lysosomes, which results in the formation of active Smo-C. Active Smo-C inactivates the kinases complex, including PKA, CK-1, and GSK-3β, and increases the formation of a transcriptional activator, primarily GLI-2A (the same occurs with GLI-3A, but lesser extent), and then GLI-2A and GLI-3A are detached from SUFU. The equilibrium between GLI-A and GLI-R results in the translocation of GLI-2A/GLI-3A, and activates the target gene in the nucleus, such as GLI-1, the transcriptional product of which acts as a transcriptional activator to amplify the Hh signaling [37]. Transcriptional target genes can also act either as positive (GLI-1*,* or negative (hedgehog-interacting protein (Hhip) and Ptch-1), creating a feedback loop for Hh signaling [38]. Ultimately, it is imperative to point out that various important signaling pathways, including the Wnt/β-catenin, TGF-β/BMP, FGF, and Notch pathways, are very closely attached to the Hh pathway, and all of them are the main participants of tissue repairing, homeostasis, morphogenesis, and organogenesis.

### 1.2. Canonical and Non-Canonical Hedgehog Signaling

**Figure 4:**
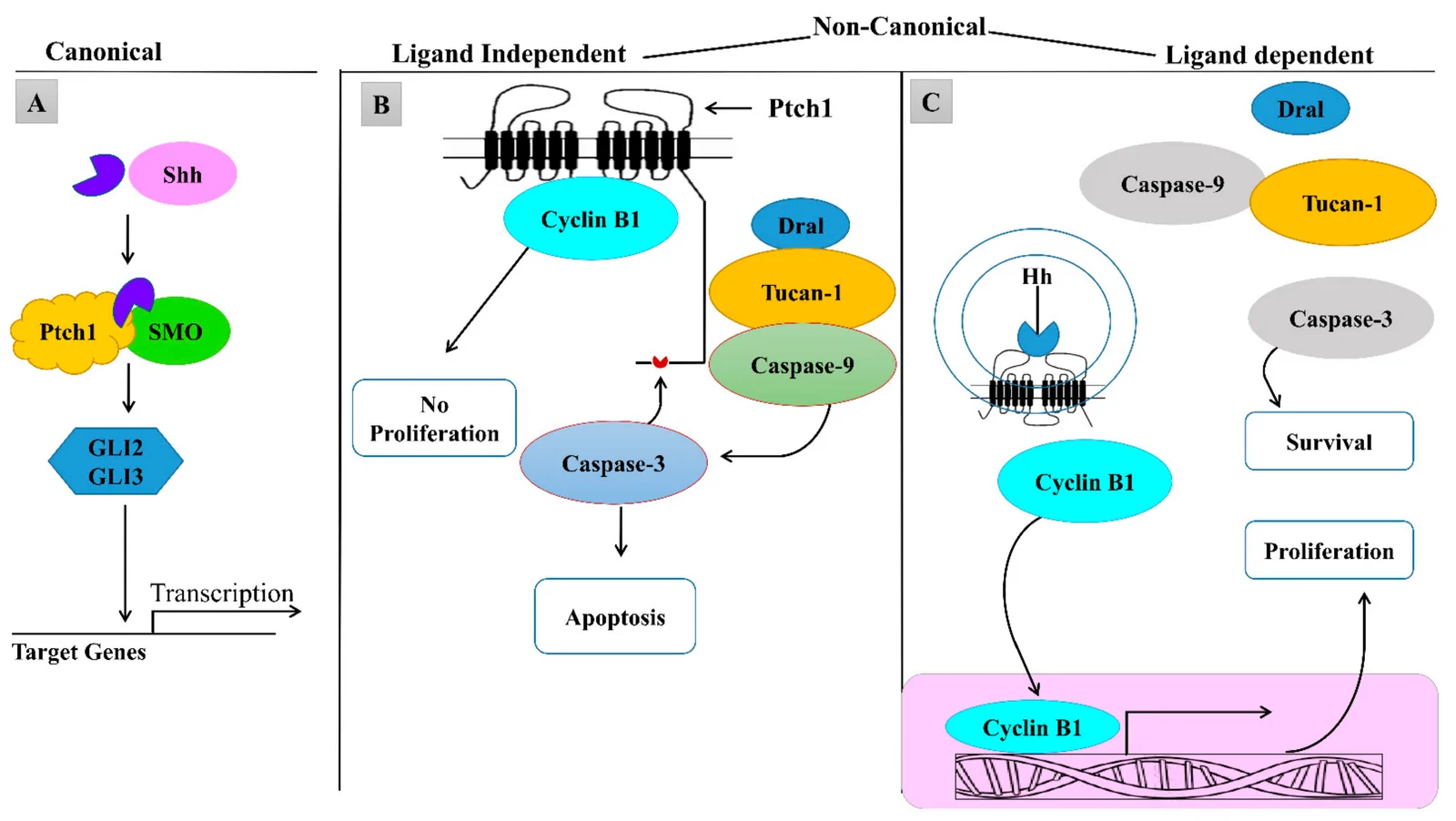
Canonical and non-canonical pathway. (**A**) In the canonical pathway, a ligand, Ptch1 or SMO activation, and transcription factors are involved. (**B**) In the non-canonical Hh ligand-independent pathway, in which Ptch1 interacts with cyclin B1 and recruits this first form of an apoptotic complex to its C-terminal domain that consists of adaptor protein Dral, the CARD-containing protein Tucan-1, and caspase-9. Caspase-9 activation is followed by caspase-3 activation, which results in apoptosis. (**C**) In the ligand-dependent non-canonical signaling pathway, the linkage of Ptch1 with cyclin B1 and the proapoptotic complex is disrupted by Hh binding, most likely due to a conformational shift in Ptch1, resulting in enhanced proliferation and survival. Red-highlighted and grey-shaded caspase shapes indicate active and inactive caspases, respectively.

The primary cilium is a basic tool for Hh signaling in nearly all cells. Canonical pathway activation is mediated by the Shh ligand and Ptch1/Smo (cell membrane molecules). It activates and stabilizes GLI transcription factors that are to be translocated to the target genes in the nucleus [27,39]. Other pathways that do not transfer their signal through Smo or GLI are known as non-canonical Hh signaling pathways. Non-canonical signaling has been categorized into two types; one is ligand dependent and the other is ligand independent. In the ligand-independent non-canonical pathway, Ptch1 interacts with cyclin B1, and a proapoptotic complex is formed including the adaptor protein Dral, the caspase-associated recruitment domain (CARD)-containing protein Tucan-1, and caspase-9. The proliferation is inhibited when cyclin B1 interacts with Ptch1 through cyclin B1 sequestering outside the nucleus. This proapoptotic complex is recruited by Ptch1 to its C-terminal domain, which results in the activation and nucleation of caspase-9. However, caspase-9 activation is driven by caspase-3, which boosts up this complex and leads to apoptosis. In this type of non-canonical pathway, the interaction between Ptch1 with cyclin B and the propapoptotic complex is impaired by the Hh pathway due to conformational changes in Ptch1, which result in enhanced proliferation and survival [40]. Canonical and non-canonical pathways’ descriptions are depicted in Figure 4.

## 2. Role of Sonic Hedgehog in Lung Development

### 2.1. Sonic Hedgehog Regulates Fetal Lung Development and Airway Repair

Sonic hedgehog (Shh), but not Dhh and Ihh, exhibits a significant role in fetal lung development (physiological) and lung repair, via activation of GLI family of transcription factors, whereas aberrations in pathway result in abnormal lung development [26]. During pseudo-glandular and canalicular stages, the Shh level is elevated at the tips of growing bronchial tubules, suggesting the contribution of Shh on the branching of lungs [27,41], while during morphogenesis, Shh is released from the endoderm and then induces mesenchymal cells differentiation and proliferation into ASMCs [42]. Shh is also well expressed in the epithelium portion during the saccular stage of embryo development and starts decreasing after birth. Thus, the expression pattern is gradually decreasing from the embryonic stage to birth [27]. Murine and human embryo development follows the similar pattern of expression of Shh [43]. Ptch-1 is significantly expressed during branching morphogenesis but decreased after birth, the same as in Shh. Smo is expressed in mesenchyme and epithelium, whereas GLI-1*,* -2, and -3 are expressed in the mesenchyme, during the pseudo-glandular stage and minimized near birth [43]. Although all GLI members are predominantly expressed in the distal mesenchyme, GLI-2 is expressed in the mesenchyme of proximal trachea and GLI-3 is present in intermediate areas between lung buds. Shh regulates the epithelial–mesenchymal crosstalk, neuroendocrine differentiation, and airway repair [44]. Shh triggers fibroblast activation and tissue fibrosis as well as stimulates ECM production [45]. In adult lung tissue, growing facts have depicted the involvement of Shh in tissue migration, as well as in the regulation of EMT-associated genes. It has also been reported that Shh is reactivated during pulmonary fibrosis and injury [46]. Thereby, data obtained from patients, suffering from idiopathic pulmonary fibrosis or non-specific interstitial pneumonia or cryptogenic pneumonia, dictate the elevated expression of Shh [47,48]. Overexpression of Shh in young mice was demonstrated to raise the collagen deposition and airway injury via fibrosis [49]. Indeed, over-expression of Shh has been detected in fibrotic remodeling after fluorescein isothiocyanate [50]. Elevation of the Shh level in the airway epithelium of a normal young mouse exhibits the proliferation and alteration of tissue structure, which is the same as in injury [51]. Shh maintains adult lung quiescence, as well as regulates the repair and regeneration process via the mesenchymal feedback mechanism [14]. Thus, Shh regulates the normal lung function during normal lung development, as well as during lung fibrosis in the adult lung [52].

### 2.2. Sonic Hedgehog Regulates Mesenchymal Proliferation and Branching Morphogenesis

Shh mediates the embryonic lung formation, by regulating the mesenchymal proliferation and branching morphogenesis. Shh knocked-out mice develop single-lobed hypoplastic lungs with diminished epithelium/mesenchyme, malfunctioning of the trachea, and trachea-esophagus fistula [53]. Deficiency of bronchial SMCs and conservation of epithelial cells phenotypes, both proximal and distal, suggest that Shh regulates the mesenchymal scaffold but not epithelial cell differentiation, which was later confirmed by probes using doxycycline-inducible surfactant protein C promoter-driven and conditional deletion of Shh [54]. Conditional deletion of Shh before ED12.5 demonstrates severe branching morphogenesis defects, whereas mild deletion of distal bronchial morphogenesis was observed after ED 12.5. Overexpression of Shh results in abnormally enlarged mesenchymal cells of the lung and respiratory failure at birth [55], pointing the involvement of Shh in differentiation and proliferation of lung mesenchyme.

Conditional deletion of Shh results in reduced proliferation of mesenchymal cells, while overexpression of Shh augments the mesenchymal cells proliferation. Both cause respiratory failure at birth, although the phenotypes are different, proposing that balanced induction of mesenchymal cells is compulsory for normal lung development and functioning. Shh also regulates the lineages of lung mesenchymal cells such as mesothelial cells entry [43,56].

GLI-2 knockout embryo dies immediately after birth, due to hypoplastic single-lobed (right lung) lungs with diminished epithelium/mesenchyme, the failure of branching supporting the role of Shh in stromal cell expansion via GLI-2. GLI-1 knockout mice that have viable normal lungs with the elevation of E-cadherin expression, and the reduction of MMP-9 and Snail expression. GLI-3-deficient mice exhibit defected lobes shape with small-sized lungs [43,57]. Interestingly, GLI-1-deficient mice exhibit normal lung function, whereas a compound mutation (single GLI-2 allele and GLI-1 deficient) results in death after birth, suggesting an overlapping of the function of GLI-1 and GLI-2 during lung development [58]. In GLI-2 and GLI-3 double-mutant mice, the most rigorous lung defects, such as failure of formation of the trachea, esophagus, and lungs, have been noticed. Double-mutant lungs, single GLI-3 allele, and GLI-2-deficient mice demonstrate abnormality in proximal lung development, such as the failure to develop tracheobronchial fistula and to distinguish the right and left lung [43]. During lung organogenesis, Shh regulates the GLI-3 processing by preventing the GLI-3 proteolysis to generate GLI-3R (repressor form); thereby, Shh-deficient lungs show an elevated level of GLI-3R.This elevation contributes to defects in differentiation and proliferation of mesenchymal cells [59]. Shh and GLI-3 double-mutant lungs exhibit increased growth potential and vasculogenesis while bronchial myogenesis is absent, as compared to Shh-deficient lungs [59], thus demonstrating that antagonistic Shh and GLI-3R gradients play a role in lung development. The lung phenotype of double mutant, GLI-2, and GLI-3 is much worse as compared to the Shh-deficient lung phenotype. The requirements of the Shh and GLI family of transcription factors, even during lung development, are mostly illustrated in Table 1. Ptch-1-deficient mice die after birth, but its role in lung development is still mysterious [34,43]. SUFU-deficient mice die after birth due to hypoplastic lungs with failure development of myofibroblasts and defective distal branching [60]. Shh-induced branching is mainly regulated by Hhip and Ptch-1, as Hhip-deficient lungs are hypoplastic [61]. Therefore, we can conclude that expression of Shh and its signaling components, but not Dhh and Ihh, is imperative for embryonic lung development and branching morphogenesis, as well as to regulate mesenchymal proliferation.

## 3. Role of Sonic Hedgehog in Asthmatic Airway Remodeling

### 3.1. Hedgehog Signaling in Tissue or Pulmonary Fibrosis

**Figure 5:**
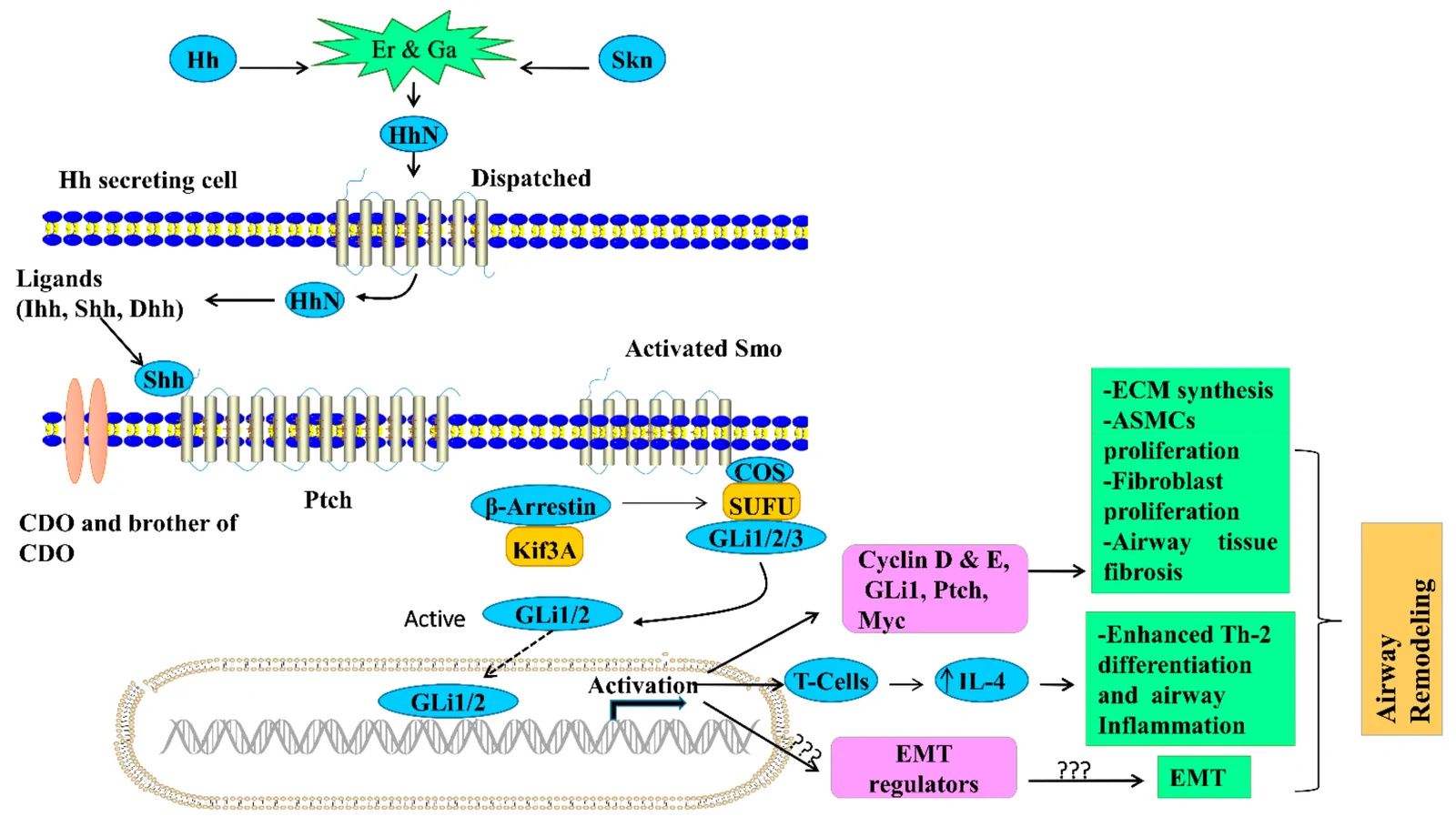
Schematic drawing of overall hypothesis and the mechanism by which Sonic Hedgehog mediates airway remodeling. In the Er or Ga, the Hh or Skn attach to the HhN. The Shh ligand attaches with Ptch through CDO and is ultimately activated the SMO and COS, SUFU, GLI-1/GLI-2 complex through β-arrestin or KIF-3A. Meanwhile, this complex activates the GLI-1/2 and translocates to the nucleus, where it activates and promotes the airway remodeling through three aberrant Hh signaling expressions—ECM synthesis and deposition, ASMC and fibroblast proliferation, and airway tissue fibrosis—which increase Th2 differentiation and inflammation and EMT synthesis. However, the exact role of Shh in airway remodeling via EMT is not yet clear (noted as???). Abbreviations: HhN, Hedgehog N-terminal domain; Skn, skinny hedgehog; COS, Kinesin-like protein costa; SUFU, suppressor of fused; KIF-3A, Kinesin family member-3A; CDO, CAM-related/downregulated by oncogenes; Smo, smooth receptor; ptch, patch receptor; Er and Ga, endoplasmic reticulum and Golgi apparatus; Hh, hedgehog; Shh, Sonic Hedgehog; Ihh, Indian Hedgehog; Dhh, Desert Hedgehog; EMT, epithelial–mesenchymal transition; IL-4, Interleukin-4; Th-2, T helper-2.

**Figure 6:**
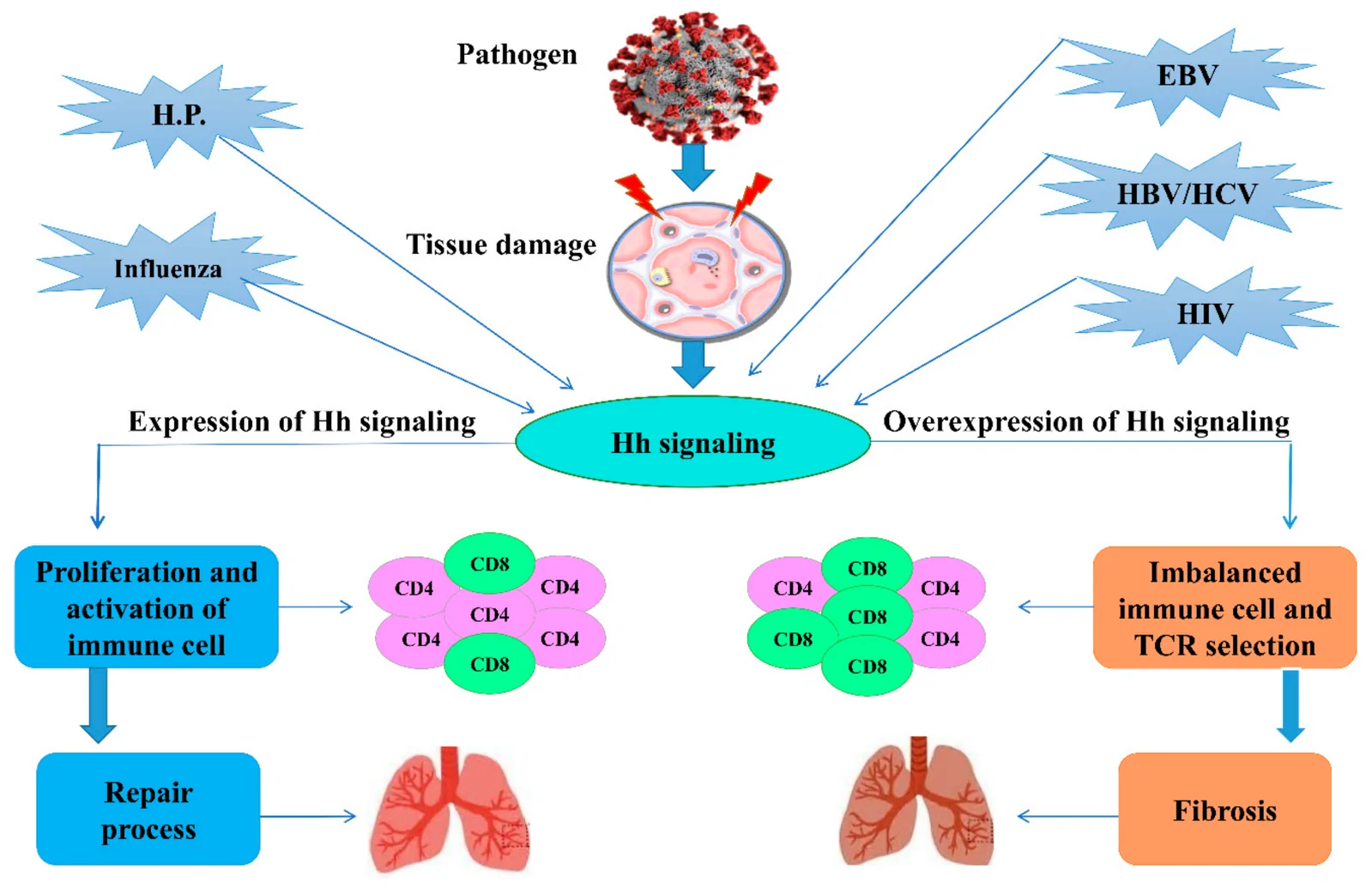
A proposed mechanism for what occurs during pathogenic and immune system interaction with the Hh signaling pathway. Pathogens become a cause to damage the host tissue and encourage the activation of the Hh signaling pathway. Pathogens play an essential role in immunity and the repair process due to key roles in. If Hh signaling is not regulated properly, it can cause fibrosis, immune cell imbalances, or T-cell receptors (TCR) selection. Pathogens such as Epstein–Barr Virus (EBV), Hepatitis B and C virus (HBV and HCV), Human Immunodeficiency Viruses (HIV), and Helicobacter Pylori (H.P.) have been shown to directly alter Hh pathway activity. Once Hh signaling is activated, it can aggravate or restrain these damaging outcomes.

Airway tissue fibrosis is a pathological state, characterized by the chronic airway inflammation, associated with the disturbance of structure and function of the airway, more precisely known as airway remodeling. Shh has a crucial role in the fibrosis of various airway structural cells, including endothelial cells, epithelial cells, and pericytes, which subsequently contribute to airway remodeling [68]. Shh is detected in fibrotic areas, such as alveolar and bronchial epithelial cells, while hedgehog-related molecules, including Ptch-1 and Smo, are expressed in the hyperplastic epithelium, fibroblasts, and interstitial inflammatory cells. Moreover, GLI-1 is observed in fibroblast, epithelial cells, and inflammatory cells (cytoplasmic and nuclear localization), whereas GLI-2 is detected in alveolar epithelial cells, primarily in a nuclear distribution, where the whole data indicate the activation of Shh signaling during lung fibrosis [26]. Hh signaling in asthmatic airway remodeling and lung fibrosis is shown in Figure 5 and Figure 6 [17]. 

During development, the Shh produces the mitogenic effect on mesenchyme and there is an association between increased Hh signaling and lung fibrosis as well as other lung diseases [45,69,70]. Idiopathic pulmonary fibrosis (IPF) is a progressive type of interstitial pneumonia with an unknown cause that is characterized by deposition of extracellular matrix and fibrotic tissues in the lungs, leading to destruction of the alveolar structures [71,72]. According to the current scenario of the IPF pathogenesis, epithelium damage causes the abnormal communication of the epithelial-mesenchymal cell, which inhibits the epithelial healing, enhances mesenchymal cell proliferation and activation, and ultimately stimulates the angiogenesis [72,73]. In idiopathic pulmonary fibrosis in human lungs, the increased expression of a lot of developmental pathway genes isexpressed by microarray data including Ptch1 [46]. The expressions of Shh are increased in the fibrotic areas of the epithelial cell lining in IPF and other interstitial pneumonia samples but are not detected in normal lungs [47,68,74]. Since the Hhip is to be considered as a direct transcriptional target for Hh signaling, the activation of Hh pathway could be decreased in COPD. This might be contributed to Hhip, which is also a negative feedback molecule in the Hh signaling pathway, and low levels of Hhip (direct effect) would be improved in Hh signaling [75]. In COPD, the Hhip protein is reduced in the lungs, suggesting an intended role for Hh signaling [76]. Gene-Wide Association Studies (GWAS) linked a locus in the vicinity of the Hhip promoter on band 4q31 to diminished lung function and COPD-related phenotypes [76,77]. A single nucleotide polymorphism is located in the Hhip promoter region that may possibly modify Hhip gene expression. 

Moreover, IPF lungs exhibit an abnormal expression of GLI-1, Smo, and Ptch1 [17,48]. The GLI-1 and Ptch1 localization are expressed during development only in mesenchymal cells. Mesenchymal and epithelial cells in fibrotic lungs suggest that the precise separation of Shh expressive and responsive cellsis lost during the process of fibrosis. Though results of gene expression patterns are conflicting during development and in disease, there is a general issue of technical limitations in examining tissues containing different cell types and gene expression levels. The outcome of fluoresce in isothiocyanate (FITC)-induced lung fibrosis is Shh overexpression in airways and alveolar epithelial cells [27]. Administration of endotracheal bleomycin in studies with the animal fibrosis model results in upregulated expression of Shh and GLI-1 in fibrotic lesions [78,79]. In GLI-1^nlacZ/+^ mice, the GLI-1-positive cells (fibroblasts and myofibroblasts) are more increased in fibrotic lesions than in uninjured lungs [52]. GLI-1-positive cells (normal lungs) are increased, which represents either the activation (de novo) of Hh-responsive cells or the production of Hh-responding cells (e.g., fibroblast proliferation). It is worth noting that the inhibition of Hh signaling at the Smo or Shh level in a bleomycin animal model fails to stop the fibrosis [52,80] and that the overexpression of Shh improved the Hh signaling during the fibrotic phase [52] revealing a potential Hh signaling role in controlling the development and survival of fibroblasts. This hypothesis was supported by in vitro study in primary lung fibroblast; the Ptch1 and GLI-1 expression was upregulated by Shh, which increased proliferation as well as rescued the lung fibroblasts from TNF-alpha-, IFN gamma-, and FasL-induced apoptosis [17]. These outcomes proposed that Hh signaling might maintain fibrosis and avoid resolution. The GLI-2/3 inhibitor that is GANT61 decreases lung fibrosis [79]. Although conservation is there due to possible unknown effects on GLI-3R, this outcome increases the possibility of ligand-independent activation of the Hh pathway in fibrosis. Evidence from other organs supports the concept that epithelium-derived Shh promotes the mesenchymal cell response in fibrotic lungs [55]. In the liver, non-alcoholic steato hepatitis and chronic cholestasis are identified by enhanced Hh signaling, which ultimately promotes the activation of the hepatic stellate cell (liver fibrosis cell) to a myofibroblastic phenotype [69,80]. However, hepatic cirrhosis and hepatocellular carcinoma are formed due to uncontrolled excessive Hh signaling [81,82]. The Hh pathway upregulates the expression of a cytokine “osteopontin” involved in wound healing, where a reduction is found in either Hh signaling or consequently in reduced fibrogenesis [83]. Thus, it was thought that there is a direct relationship between GLI and GLI-binding sites in the osteopontin promoter. Additionally, liver fibrosis is mostly caused by HBV and HCV infections, which are mediated by active Hh signaling [84]. In the kidney, a myofibroblast with active Hh signaling is responsible for urethral obstruction-induced fibrosis and is blocked by the Smo antagonist ion GLI-1^−/−^ in mice [70,85]. Information about Hh signaling in human lungs diseases is documented in Table 2.

### 3.2. Hh Signaling in Inflammation, COPD, and Asthma

The airway epitheliums are regularly injured by trauma, contaminants, and pathogens, resulting in a wide range of inflammatory, immunological, and defensive responses. To restore lung homeostasis after damage, a rapid wound-healing regenerative system is intended. Hh signaling may play an effective role in epithelial damage and repair by influencing proliferation and the immune response [44]. Meanwhile, the Hh upregulation has three possible actions: (a) it causes Th2 inflammation in mice and human allergic asthma through sending a signal to activate Th2 differentiation and cytokine production in CD4 T-cells that leads to Th2 inflammation; (b) various responses to the Shh signal are due to tissue-dependent, underlying changes in the composition of differentiation and stimulation of inflammatory and immune cells present in the lung caused by damage; (c) Shh signaling is combined with other environmental factors, as well as tissue responses, to regulate whether Shh produces anti-inflammatory or pro-Th2-inflammatory effects. However, Hh upregulation may be seen as a natural inflammatory process in terms of maintaining airway homoeostasis, while abnormal Shh expression and Hh signaling pathways can also lead to the formation and development of Th2 inflammation and chronic inflammatory lung diseases [86]. An in vivo study revealed that exposure of mice to house dust mite allergens exhibited the elevation of Shh expression, owing to CD_4_+T-cell. This augmentation of Shh signaling results in overproduction of interleukin (IL-4) and over-responses of Th-2, which aggravate allergic inflammatory lung disease [87]. Airway diseases, such as asthma and COPD, are linked with altered Hh signaling, as shown in Table 2. As a consequence of chronic severe inflammation, both diseases express peribronchial fibrosis and tissue remodeling, along with all those processes involved in fibroblast growth and matric deposition, parallel to lung fibrosis [88,89]. 

Allergic asthma is a chronic airway inflammatory disease characterized by inflammatory cells, such as mast cells, basophils, eosinophils, Th2 cells, and innate lymphoid cells interacting with airway epithelial cells, resulting in inflammation, increased mucus production, bronchoconstriction, and tissue remodeling [90]. Several studies have also suggested that the Hh signaling pathway is involved in the interaction between inflammatory and respiratory cells that leads to allergic asthma. Moreover, CCL2, a major chemokine for the upregulation of leukocytes, and the recruitment of leukotriene C4 released into the airway was induced or inhibited by Shh recombinant treatment or Smo inhibitor treatment in epithelial and leukocyte cells, respectively, making a contribution to airway hyperresponsiveness [88,91]. Another GWASresearch study demonstrated that linkage between Ptch1 and the Hhip region decreases the performance of the lungs, worsening the asthma-related remodeling [75]; however, the study is uncertain how the Hh pathway can contribute to the pathophysiology of asthma. There is one possibility that the Hh signal is received from airway epithelial cells by CD4 T-cells, which plays an essential role in airway inflammation, and there is a need for Hh signaling in the thymus for normal differentiation [87,92]. Inflammatory lung diseases support the contribution of the overexpression of Th-2 and interleukin in asthmatic airway remodeling [93,94]. Allergic inflammation of airways, as well as the disturbance of the neuronal control of airway smooth muscle, contributes to the pathogeneses of asthma. MiR-206/Shh/brain-derived neurotrophic factor (BDNF) coordinates innervations and the formation of airway smooth muscles [95].

Shh inhibits miR-206 expression resulting in the elevation of BDNF, which augments and supports the effect of ASMCs proliferation and airway hyper-reactivity in asthmatics [20,24,96,97]. Even though the expression of Shh by airway epithelium encourages the Th2 differentiation of CD4 T-cell, the allergic response might be exacerbated in a dust mite-induced asthma model. Allergic response in an ovalbumin (OVA)-induced asthma model was not altered by CD4-specific gene deletion of Ptch1, which points out the activation of the non-canonical Hh pathway in CD4 T-cells [27]. Besides, lymphocytes donot possess primary cilia, while Hh signaling may [98]. Another possibility like epithelial Shh signaling to peribronchial stromal cells (fibroblast and pericytes) with a subsequent plan for tissue remodeling is also valid. In the healthy adult murine lung, the Hh-responsive cells are situated in the interstitial spaces around airways and vessels [52,84]. Hh signals are also responded to by Clara cells, eosinophils, and lung epithelial cells, possibly related to the pathogenicity of allergic illness. In mouse models of airway allergic disease, Hh pathway inhibition via Smo inhibition or therapeutic interventions with a Shh-neutralizing antibody was shown to reduce Th2 responses, recruitment of the cells to the lung and BAL, and mucous production, as well as aberrant airway remodeling [86]. The use of inhaled glucocorticoids such as budesonide can alter the localization of Smo together with Hh activity; thus, sensitivity is increased towards Hh ligand input in asthma and COPD [99]. Taken together, it can be proposed that aberration of Shh signaling contributes directly and/or indirectly to airway remodeling, but insight philosophy is still enigmatic, requiring further study for better defining its role.

### 3.3. Sonic Hedgehog in ECM Production and EMT

In skin lesions with systemic sclerosis, patients express the overexpression of Shh and abnormal Hh signaling. Shh triggers collagen production and ECM-related proteins, either by fibroblast activation or by encouraging the transformation of fibroblaststo myofibroblasts (Figure 5) [45]. While in asthmatics, there is a dense deposition of the collagen network, instead of loose distribution, which causes lung physiology to be disrupted [100]. Moreover, pulmonary fibrosis and epithelial/mesenchymal crosstalk, in the case of non-small-celllung cancer (NSCLc), mediated by Shh, result in an elevated production ofECM-related proteins, such as fibronectin and fibroblast collagen [17,101], which reflects the indirect involvement of Shh in airway remolding (Figure 5) [102]. In a model of adult lung injury, an elevated level of GLI-1-rich mesenchymal cells has been observed in alveolar septa and fibrotic lesions, while increased ECM production has also been reported by the adenovirus-mediated overproduction of Shh [52]. In cases of murine lung inflammation, human idiopathic pulmonary fibrosis, and fluorescein isothiocyanate-induced fibrosis, an elevated level of Shh has been reported [68]. Reports also suggest that Shh elevates the proliferation of fibroblasts and production of ECM, and it has already been reported that Shh boosts the ECM production and induces the transformation of fibroblasts to myofibroblasts [45]. Hence, such findings support the possibility that Shh is involved in subepithelial fibrosis in asthmatic airways, which is caused by an imbalance between ECM degradation and deposition, resulting in airway remodeling [103]. Due to interaction between Hh and TGF/Smad signaling cascades, Shh is implicated in epithelial–mesenchymal transition (EMT) during the developmental phase, as well as in adult tissue [26,48,104,105], suggesting that EMT is involved in airway remodeling (Figure 5) [106,107,108]. Dermal overexpression of Shh and treatment of bleomycin cause skin fibrosis in mice, which is blocked by Smo siRNA and the Smo inhibitor LDE223. Ptch1 ½ mice are more vulnerable to bleomycin-induced skin fibrosis and also elevated the Hh signaling [45,109]. Topical gene treatment can be used for skin wounds, such as DNA encoding that increases wound repairment. Whereas corresponding studies demonstrated that, upon closure of a wound, proliferation and vascularization were suppressed at wound sites via intraperitoneal administration of cyclopamine, an effective smoothened antagonist [84].

## 4. Role of Hedgehog Signaling as a Pathogen Target and Used in Wound Repair, Immune System Pathway, and Immune Diseases

### 4.1. Hedgehog Signaling Used as a Pathogen Target

The Hh signaling mostly occurs in embryogenesis, but it also occurs postnatally in the lungs, gut, hemopoietic, skin, immune system, and various other tissues. Postnatal signaling has played an effective role in maintaining the stem cell and tissue homeostasis as well as regulating the lymphopoiesis and hematopoiesis. It also has been stated that the Hh signaling showed their activation in response to pathogen, damaging, and wounds agents. Thus, Hh signaling has been shown to be a target of various pathogens [84].Here is some examples of Hh signaling that interact with such pathogens.

Hepatitis B (HBV) and Hepatitis C (HCV) are the main causes of liver cirrhosis and hepatocellular carcinoma. The infected liver from HBV and HCV patients demonstrated that an excessive production of Hh ligands in hepatocytes and Hh-responsive cells accumulation at higher levels have more dire outcomes [110,111]. It was confirmed by further complementary studies that in vitro treatment of liver cells with entire replicon HBV and serum from HCV patients improved the targets for Hh in a GLI-dependent method and led towards pro-fibrotic effects [112,113]. The viral protein of HBV increases the stability of the GLI protein and promotes nuclear accumulation, but it remains unclear to promote infection through such viral activities. Moreover, in liver cells, increasing Hh signaling promoted HCV replication, suggesting that there is a positive feedback relationship between Hh pathway activation and viral replication [84]. Increased Hh target genes have been observed inepithelial cells infected with Epstein Bar Virus (EBV). It was indicated that significant increase in GLI-1 expression caused by EBV was in parallel to a significant drop in the expression of human leukocyte antigen that is engaged in demonstration of viral antigen to cytotoxic T-cells, and hence may possibly restrict the EBV detection through the immune response [114,115]. Figure 6 demonstrates how the Hh pathway targets some of the pathogens and plays a role in wound repair and immune system pathway diseases.

A Gram-negative bacterium “Helicobacter Pylori (*H. pylori*)” is prevalent in the stomach and is considered as the main cause of gastric cancer and gastric atrophy [116]. For the periods of early *H. pylori* infection, the Hh upregulation was shown in parietal cells (secreting HCL) in vivo (mouse model) as well as in vitro (growth of epithelial cell culture from dissociated gastric glands). The upregulated signaling appeared due to inflammation or repair process as well as recruitment of macrophages to the infection site along with NFκB induction. It was analyzed that prolonged exposure to *H. Pylori* in the gastric epithelium, together with the depletion of parietal cells (secreting HCL) in the digestive glands, resulted in a significant reduction in Hh expression in both *H. Pylori* animal models and chronic gastritis *H. Pylori*-positive patients [84]. Hh expression could be restored by eradicating *H. Pylori* infection, and Hh signaling could be reduced by macrophages secreting regulatory cytokines [117]. However, it has been shown that *H. pylori*-dependent protein upregulation (caudal-type home box 2; CXD2) is capable of blinding the Shh promoter and inhibiting Hh pathway expression [118,119,120]. These findings show that expression of Hh target genes is not activated directly by the viral proteins; rather, canonical signaling activates Hh signaling first, and subsequently, a viral interaction mediates to change the pathway consequences.

### 4.2. Hedgehog Signaling Regulates Wound Repair

Pathogenic interactions with more centrally controlled, linked host proteins are usually established to ensure effective use of respective genomes, allowing for multiple processes to be targeted simultaneously. The interface residues of these virulence factors are constantly evolving, either to avoid or improve their ability to bind to their host proteins. As a result of its important function in a variety of activities, including wound healing and immunology, certain viruses have figured out how to regulate Hh signaling [121]. An important role of Hh signaling has been established in wound healing for several types of cells such as lung, pancreas, and skin wherever damaged tissue is associated with Hh activation [84]. As Hh signaling plays a major role in remodeling and tissue homeostasis, we hypothesized that the Hh pathway might be triggered as a defense mechanism to control the infection and promote tissue repair. It was observed that naphthalene damage induced Shh signaling in lung epithelial cells, which was very related to the stimulation of fetal expansion [44]. Shh is also elevated in lung homogenates during hyperoxia-induced injury as well as healing [122]. The same occurs with bleomycin administration [79], and during impaired airways in lungs exposed to fluorescein isothiocyanate for a long time.

In the skin, in vitro increased permeability of cultured skin cells with the Hh ligand boosted the expression of angiogenic signals, accelerated fibroblast production, and increased adhesion, migration, and filament formation of endothelial progenitor cells to facilitate wound healing [123]. Likewise, topical gene therapy on wounds utilizing DNA expressing Shh improved wound healing, while parallel experiments found that intraperitoneally injected cyclopamine, a powerful smoothened antagonist, hindered wound closure, angiogenesis, and growth at wound sites [123,124,125,126].

Tissue fibrosis, which is characterized by the production of harmful scar tissue, can occur when repair processes fail. Unsurprisingly, a substantial relationship between Hh signaling tissue fibrosis in various tissues is affected by chronic diseases. Most of it is due to the H-dependent stimulation of epithelial to mesenchymal transition cells, resulting in the increased deposition of the extracellular matrix [38]. Pulmonary fibrotic diseases, such asIPF, nonspecific interstitial pneumonia, and interstitial lung diseases [47,84,127,128], are described by the Shh expression and Ptch1 in the fibrosis region. In bleomycin-induced fibrosis, GLI-1^nlacZ/+^ mice have a higher number of Hh-responsive cells, suggesting that overexpression of Shh can exacerbate impairment [52].

The Hh ligand, which is expressed by liver cells in cellular injury, induces thecellular differentiation of fibroblasts and myofibroblasts, which is essential for normal liver regeneration. Excessive signaling, as seen in the lungs, causes fibrosis, which, if not regulated, can lead to cirrhosis and HCC. In a liver fibrosis model induced by nonalcoholic steatohepatitis Hh induces the overexpression of osteopontin (OPN), a cytokine implicated in tissue repair or healing, reducing either Hh signaling or diminished OPN. GLI and GLI-binding sites were assumed to have a strong link with the OPN promotor. Furthermore, infections such as HBV and HCV are also known for causing liver fibrosis, which is thought to be mediated by increased Hh signaling [84].

### 4.3. Role of Hh Signaling in Immune System Diseases

The Hh pathway performs an essential function in immune system, which is conserved from flies to human, although the relevant signaling output and how it acts as a defense system differ between invertebrates and vertebrates. A study was designed in Drosophila in which Hh signaling established a first-line protection in the gut against detrimental uracil-secreting pathogens by provoking microbicidal reactive oxygen species (ROS) production [129,130,131]. The expression of flies was underway when the bacteria were infected, and the RNA directed to many Hh pathway (canonical) components resulted in a lower production of ROS and a high death rate [84]. Subsequent experimental studies determined that Cadherin 99C (Cad99C) can save this phenotype, and Cad99C-dependent signaling endosomes stimulated the dual oxidase enzyme (DUOX), which consequently stimulates ROS production to liberate phenotype in response to the detection of uracil. The secretion of the Shh ligand is carried out from epitheliocytes to the T-cell progenitor that alters cell lineages as well as production [132].In mammals, Hh signaling has many directional approaches to determining the immune system activity by regulating a variety of T-cell factors, such as growth, separation, and function [92,132]. Pro-thymocytes eventually mature into either cytotoxic T-cells or helper cells, as indicated by CD4 or CD8 expression on cellular receptors, after going through a series of differentiation steps. During this whole process, the development of thymocytes starts from a double-negative expressing marker to double-positive markers along with a lot of intermediate steps (CD25 and CD44), then particular matured positive cells are able to leave the thymus into the bloodstream [84]. Besides, the selection and maturation of the T-cell receptor are carried out through gene variants that could identify foreign peptides [133]. In a mouse model, the thymocyte investigational phenotype demonstrates that the Hh signaling pathway is essential for the management of T-cell activation. It also controls the supply of TCR selection by determining the CD4 vs. CD8 final differentiation phase, regulating the conversion from double-negative to single-positive cells, and attenuating TCR signaling strength [84]. Direct stimulation of immune cells (cytokine expression-secreted factors) that can produce an effect on alternative cells is an additional potential of the Hh pathway. The CD4 T-cell (peripheral) could counter Hh signaling through following the activation of TCR due to retaining expression activity of Hh pathway [127,128]. Hh signaling can also stimulate the clonal development of the previously mentioned cell population by boosting the cytokine (interleukin-2, 10) and interferon (IFNγ) expression, which encourages the entrance into the S-G2 phase of the cell cycle. Moreover, the stimulation of in vitro human macrophages with recombinant Shh upregulates some cytokines (IL-6, -8, and -10), and chemokine expressions as well as Monocyte Chemoattractant Protein-1 (MCP-1), but the deficiency was found in other expressions of the conditional Hh knockout model of *H-pylori* infection [134,135]. Shh also stimulates the pro-fibrotic cytokines’ expression, such as IL-13 and IL-4 expression, during liver fibrosis in natural killer T-cells. Besides, Hh regulates the proliferation of immune cells along with cytokine stimulation. There are also some leukemias and autoimmune disorders that are linked to the activation of an irregular pathway. For example, allergic asthma is to be considered an autoimmune condition wherever the underlying pathophysiology is linked to an aberrant immune response to an inhalational aeroallergen. In the present disease, activation of the pathway results in an oversupply of naïve T-cell conversion to Th2 cells (T helper 2). This is accomplished by increasing the production of several Hh-related genes that identify Th2 cells, such as cytokines like IL-4 [84]. Hence, even as Th2 cells provide defense in opposition to extracellular parasites as well as tissue remodeling that occurs as a result of injury, it also plays an important role in allergic and inflammatory disorders [136]. Likewise, influenza infection actively induces the cytokines’ expressions, like IL-6 and CXCL-10, and there is also a cell-autonomous relationship between influenza NS1 and Hh signaling [137]. It was noted that IL-6 was comparatively higher in those animals infected with a virus-carrying (A122) point alteration in NS1; hence, we hypothesize that past influenza pandemic was produced due to excessive production of cytokines storms (Hh dependent) [138]. Taken together, these studies specify that a selective population of immune cells respond to the Hh signal, and consequently increase proliferation as well as upregulation of cytokines. Hh signaling increasingly occurs in HBV, HCV, EBV, and HIV infections that might assist the virus in avoiding the reaction of immune pathway by disrupting the immune system balance (Figure 6). In contrast, the activation of pathway throughout influenza infection might restrain the immune reaction to escape infection and provide safety against cytokine storms [84].

## 5. Potentially Therapeutic Significance

The ultimate targeting of canonical/non-canonical Hh pathways at downstream levels can inhibit fibroproliferative disorders, mainly tumors, but these antagonistic agents still need to be evaluated in asthmatic animal models to ensure that the physiological participation of Ptch, Smo, GLI, and other factors in the Hh pathway will open broad research avenues for the remedy of asthmatic airway remodeling and immune diseases. Airway tissue fibrosis, ASMC proliferation, airway hyper-reactivity, and overexpression of collagen, fibronectin, ECM, mesenchyme, and IL-4 are all hallmarks of Shh overexpression and/or aberrant signaling at various levels; all of these actions are similar to be seen in airway remodeling.

Shh expression is upregulated by JAK/STAT6 signaling triggered by IL-4/13, which promotes allergic airway epithelial remodeling. Whereas, in animal models with ovalbumin- or house dust mite allergen-induced airway inflammation, intratracheal suppression of STAT6 by AS-1517499 dramatically reduced not only the allergen-induced Shh overexpression, but also the hyper-responsiveness and goblet cell metaplasia [23]. Moreover, in the pathophysiology of COPD, the hedgehog interacting protein (HHIP) may have shielded the lungs from airway remodeling by suppressing the hyperproliferation of ASMCs, which is linked to metabolic change to aerobic glycolysis [139]. For the appropriate therapeutic purpose, it is compulsory to stop the overexpression and/or aberrant signaling of canonical/non-canonical hedgehog pathway at the downstream level by using potentially safe and effective antagonists, such as cyclopamine (Smo antagonist) [44,140] and SANT 1-4 and KAAD-cyclops and CUR-61414 (Smo and Shh antagonists) [124], but all of these are still under study.

As previously described, Smo is overexpressed during hyperplastic epithelium, fibroblasts, and interstitial inflammatory cells’ activation. Cyclopamine, a specific Smo antagonist [124], is widely considered for the treatment of cancer [141], but it still needs to be explored more for the treatment of asthmatic airway remodeling. Downstream altered Hh signaling could be a challenge for Smo-targeting therapies. Protein kinase-A agonist, such as forskolin, keeps the GLI in an inactive state by antagonizing its effect [142]. Similarly, antisense oligonucleotides (synthetic miRNAs) targeting GLI-1, a transcriptional factor, antagonizes the GLI-mediated activity [143]. It has also been reported that direct inhibition of GLI-dependent transcription is recommended as a potential pharmacological approach for the IPF treatment and also for indications relevant to airway remodeling [48]. Recently, the Hh pathway was recognized as an essential component in COPD and GLI-2 as a key player during airway epithelial cell differentiation. SMO and GLI-2 were identified as possible epithelial remodeling markers in COPD patients. The GLI-2 location in the airways could be used to express the status of COPD patients and aid in the development of novel treatment to combat epithelial remodeling as well as to enhance respiratory function [144]. Thus, systemic pharmacological smoothened inhibition in a mouse model of allergic airway illness reduced the infiltration of T-cells and differentiation of Th2 cells in the lung. Moreover, it attenuated the inflammation, disease severity, Mucin gene Muc 5ac expression, andserum IgE, and it seemed to be defensive against lung diseases [145].

Various signaling pathways, such as TGF-β, Notch, and Wnt/β-catenin, develop complex interactions with Shh during pulmonary fibrosis. The Shh pathway attenuated the expression of collagen-1 and fibronectin, while blocking the TGF-β effect on these proteins [26,146], indicating Shh/TGF-β crosstalk. Shh regulates TGF-β-dependent myofibroblastic differentiation in idiopathic pulmonary fibrosis [48]. Likewise, Shh expression has increased in Wnt-5a null mice, suggesting that targeting Wnt signaling causes abnormal branching morphogenesis [147]. Smoke activates the Hh and Wnt pathways in bronchial epithelial cells, resulting in airway remodeling and tumor growth. Therefore, treatment with sulindac (Wnt inhibitor) attenuates the tumor mass in mice [148], but Hh inhibitors still need to be studied regarding airway remodeling.

Furthermore, different recent FDA-approved treatments, such as CDC-0449 and LDE225, inhibit SMO, but arsenic trioxide inhibits GLI-1/2. As a result, it makes perfect sense that FDA-approved medicines that suppress Hh signaling could be used as powerful inhibitors to treat a variety of pathogenic illnesses. Despite currently existing medicines, such as antivirals and vaccinations, which target specific viral proteins that rapidly mutate, therapies that target host targets could provide stronger protection over a wide range of strains [84]. Remarkably, Hh antagonists and agonists seem to be effective for the treatment of HIV-infected cells and tissues. GLI antagonist were shown to reduce HIV-dependent cellular proliferation and migration, while smoothened agonists strengthened the blood–brain barrier, reducing leukocyte influx into the brain, and therefore restraining the viral niche into humanized mice [149,150,151]. These variations highlight ascenario in which the site of infection can be regulated by different Hh-modulating systems. Thus, current HIV investigations emphasize the significance of precisely choosing appropriate treatment, because the physiological processes mediated by this pathway are costly and exceedingly complicated.

This may require a synergistic paradigm where numerous medications are targeted to various Hh-dependent processes, allowing for signaling to be strengthened or reduced in a context-dependent manner. This could be accomplished by combining medications that target signaling pathway elements that have beneficial and harmful effects. Furthermore, establishing the suitable technique by which viral elements link with the pathway would almost certainly limit the investigation into effective treatments.

From these data, it can be inferred that Hh signaling regulates the indications associated with airway remodeling and immune system pathway diseases either directly or through other pathways; thereby, a combination of genetic/personalized and pharmacological analysis of this pathway will provide an efficient approach in future research.

## 6. Conclusions

To date, many research investigations have been published to investigate the role of Hh signaling in numerous airway diseases, but clinical evidence to confirm the role of the Hh signaling pathway is still lacking in asthmatic subjects. The facts and figures of experimental animal data support the pivotal role of Hh signaling in embryonic lung expansion, initiation, and the development of asthmatic airway remodeling, which is linked with impaired early lung development with asthmatic remodeling. Moreover, Hh signaling acts as a target for pathogens, and many Hh antagonists have been identified that might be effective in the treatment of wound repair and pulmonary fibrosis through overcoming the aberrant expression and overproduction of Hh signaling. We postulate that the significant postnatal contribution in wound repair and immune response disease is a key factor for pathogenic control. These findings could lead to new therapies for asthmatic airway remodeling, lung injury, and immune system pathway diseases involving Hh-stimulatory/inhibitory drugs or combined medications (personalize/genetic and pharmacological) that target signaling aspects that have both beneficial and adverse effects, which interrupt/reverse these connections, suggesting a promising new research direction.

## Figures and Tables

**Table 1:** Hedgehog signaling pathway gene knockout for lung phenotypes.

Genotype	Phenotype	Remarks	Refs.
Shh^−/−^	Hypoplastic lungs (single lobe) with diminished epithelium; trachea and trachea-esophageal fistula to be malfunctioned.	Lethal at birth	[53,62]
Shh^+/−^	There is not any reported abnormality.	Not lethal	[63]
Ptch1^−/−^	It is lethal before the beginning of lung development.	Lethal at E8.5–E9.5	[34,64]
Ptch1^+/−^	There is not any reported abnormality.	Not lethal	[64]
GLI-1^−/−^	Normal appearance.	Not lethal	[58]
GLI-2^−/−^	Hypoplastic lungs along with severe patterning abnormalities in single-lobed right lung and decreased mesenchyme, a little hypoplastic trachea and esophagus	Lethal at birth	[65]
GLI-2^+/−^	Normal appearance.	Not lethal	[58]
GLI-3^−/−^	Hypoplastic lungs with reduced size and abnormal appearance of lobes.	Lethal at E14.5	[66]
GLI-3^+/−^	Normal appearance.	Not lethal	[67]
GLI-1^−/−^, GLI-2^+/−^	Hypoplastic lungs with diminished size and less severe than GLI1^−/−^; GLI2^+/+^	50% lethal until P21	[58]
GLI-1^−/−^, GLI-2^−/−^	Two lobes are severely hypoplastic	Lethal at birth	[34,58]
GLI-2^−/−^, GLI-3^+/−^	Hypoplastic lungs with development of abnormal proximal lung that is failed to perform left and right lung separation, consequently, tracheobronchial fistula and distal lung partially formation occur	Lethal at birth	[65]
GLI-2^−/−^, GLI-3^−/−^	Severe form of phenotype and shows failure performance in order for the formation of the trachea, lung, and esophagus	Lethal at E10.5 but some embryos survive until E13.5	[65]
Hhip^−/−^	Hypoplastic lung (single lobe) with abnormal formation of the second generation of lung buds	Lethal at birth	[61]
Hhip^+/−^	There is not any reported abnormality.	Not lethal	[61]
Hhip^−/−^, Ptch1^+/−^	It is a form of hyoplasia more severe than Hhip^+/−^ lungs; thickened epithelium.	Lethal at birth	[61]

**Table 2:** Hedgehog signaling in human lung diseases.

Disease Type	Technique	Outcome	Refs.
**Parencymal lung disease**			
IPF	Microarray	Gene expression of Ptch1 is altered in IPF lungs disease.	[46]
	ISH	Shh gene is highly expressed in the epithelium of the fibrotic zone	[48,74]
	IHC	Smo, GLI1, and Ptch1 are expressed in the fibroblastic zone of IPF lung disease.	[17]
	IHC	Shh is expressed in hyperplastic alveolar type II cells in the fibrotic area.	[47,68]
	qRT-PCR	Shh, GLI1, and Ptch1 are upregulated in IPF lungs disease.	[17]
	ELISA	Shh is high in BALF from IPF lungs.	[48]
NSIP	IHC	Shh is expressed in epithelial cells of the thickened alveolar wall.	[47,68]
	ISH	Shh is minimally expressed in the epithelium but higher than in normal lungs.	[74]
COP	IHC	Shh is expressed in buds of organizing exudates.	[47]
**Airway diseases**			
Asthma	GWAS	SNPs in Hhip regions on 4q31 and Ptch1 on 9Q22-31 is associated with decreased lung function (FEV1/FVC ratio) and asthma-related phenotypes.	[75]
COPD	qRT-PCR	Hhip is decreased in COPD lungs disease.	[76]
	WB	Hhip is decreased in COPD lungs disease.	[76]
	GWAS	SNP in region close Hhip gene on 4q31 related to diminished lung function (FEV1/FVC ratio) and COPD-related phenotypes.	[76,77]

Abbreviations: BALF—bronchoalveolar lavage fluid; COP—cryptogenic organizing pneumonia; COPD—chronic obstructive pulmonary disease; GWAS—gene-wide association study; IHC—immunohistochemistry; IPF—idiopathic pulmonary fibrosis; ISH—in situ hybridization; NSIP—non-specific interstitial pneumonia; qRT-PCR—quantitative reverse transcription polymerase chain reaction; SNP—single-nucleotide polymorphism; WB—Western blot.

## Data Availability

Not applicable.
