# Correction: Zeng et al. Hedgehog Signaling: Linking Embryonic Lung Development and Asthmatic Airway Remodeling. *Cells* 2022, *11*, 1774

**DOI:** 10.3390/cells14050356

**Published:** 2025-02-28

**Authors:** Ling-Hui Zeng, Muhammad Qasim Barkat, Shahzada Khurram Syed, Shahid Shah, Ghulam Abbas, Chengyun Xu, Amina Mahdy, Nadia Hussain, Liaqat Hussain, Abdul Majeed, Kashif-ur-Rehman Khan, Ximei Wu, Musaddique Hussain

**Affiliations:** 1 Department of Pharmacology, Zhejiang University City College, 51 Huzhou Street, Hangzhou 310015, China; 2 Key Laboratory of CFDA for Respiratory Drug Research, Department of Pharmacology, School of Medicine, Zhejiang University, Hangzhou 310058, China; 3 Department of Basic Medical Sciences, School of Health Sciences, University of Management and Technology Lahore, Lahore 54000, Pakistan; 4 Faculty of Pharmaceutical Sciences, Government College University, Faisalabad 38000, Pakistan; 5 Medical Pharmacology Department, International School of Medicine, Istanbul Medipol University, Istanbul 34000, Turkey; 6 Department of Pharmaceutical Sciences, College of Pharmacy, Al Ain University, Al Ain 64141, United Arab Emirates; 7 Faculty of Pharmacy, Bahauddin Zakariya University, Mulatn 60000, Pakistan; 8 Faculty of Pharmacy, The Islamia University of Bahawalpur, Bahawalpur 63100, Pakistan

In the original publication [1], there was a mistake in Figure 1 as published. Figure 1 was found to include the unauthorized use of content from another Figure 1, published in another paper [2]. The corrected Figure 1 appears below. The authors state that the scientific conclusions are unaffected. This correction was approved by the Academic Editor. The original publication has also been updated.

## Figures and Tables

**Figure 1:**
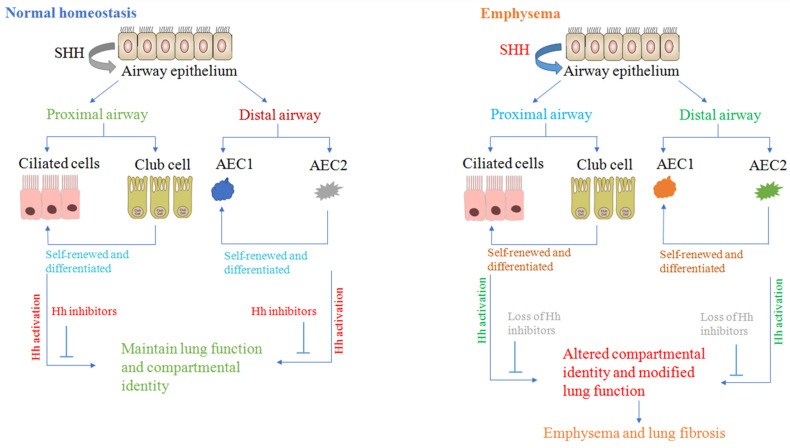
Asymmetric hedgehog activation model that maintains distinct compartmental identity. We propose that differential Hh activation is a promising mechanism for maintaining compartmental-specific identity as well as function in the lungs. Endogenous inhibitors of Hh activation may be lost, disrupting the physiological asymmetry of Hh and resulting in altered compartmental identity and structural changes found in lung disorders.
