# Correction: Zeng et al. Hedgehog Signaling: Linking Embryonic Lung Development and Asthmatic Airway Remodeling. *Cells* 2022, *11*, 1774

**DOI:** 10.3390/cells15161500

**Published:** 2026-08-20

**Authors:** Ling-Hui Zeng, Muhammad Qasim Barkat, Shahzada Khurram Syed, Shahid Shah, Ghulam Abbas, Chengyun Xu, Amina Mahdy, Nadia Hussain, Liaqat Hussain, Abdul Majeed, Kashif-ur-Rehman Khan, Ximei Wu, Musaddique Hussain

**Affiliations:** 1 Department of Pharmacology, Zhejiang University City College, 51 Huzhou Street, Hangzhou 310015, China; 2 Key Laboratory of CFDA for Respiratory Drug Research, Department of Pharmacology, School of Medicine, Zhejiang University, Hangzhou 310058, China; 3 Department of Basic Medical Sciences, School of Health Sciences, University of Management and Technology Lahore, Lahore 54000, Pakistan; 4 Faculty of Pharmaceutical Sciences, Government College University, Faisalabad 38000, Pakistan; 5 Medical Pharmacology Department, International School of Medicine, Istanbul Medipol University, Istanbul 34000, Turkey; 6 Department of Pharmaceutical Sciences, College of Pharmacy, Al Ain University, Al Ain 64141, United Arab Emirates; 7 Faculty of Pharmacy, Bahauddin Zakariya University, Mulatn 60000, Pakistan; 8 Faculty of Pharmacy, The Islamia University of Bahawalpur, Bahawalpur 63100, Pakistan

In the original publication [1], there was an error in Figure 1. The published figure inadvertently included unauthorized use of content derived from Figure 1 of a previously published article by Wang et al. [2]. Although the figure has been redrawn, its conceptual design and overall visualization were adapted from the work of Wang et al. (*J. Dev. Biol.* **2019**, *7*, 14) [2] and should have been appropriately acknowledged. Accordingly, the legend of Figure 1 has been revised to include the statement: “Adapted from Wang et al., *J. Dev. Biol.* 2019, 7, 14 [4].”. The corrected Figure 1 caption appears below. The authors apologize for this oversight and confirm that the scientific conclusions of the article remain unaffected. This correction was approved by the Academic Editor, and the original publication has been updated.

**Figure 1.** Asymmetric hedgehog activation model that maintains distinct compartmental identity. We propose that differential Hh activation is a promising mechanism for maintaining compartment-specific identity as well as function in the lungs. Endogenous inhibitors of Hh activation may be lost, disrupting the physiological asymmetry of Hh signaling and resulting in altered compartmental identity and the structural changes observed in lung disorders. Adapted from Wang et al., *J. Dev. Biol.* 2019, 7, 14 [4].
